# PLAR: a combined percutaneous and arthroscopic treatment for iliotibial band syndrome description of surgical technique and short-term results: description of surgical technique and short-term results

**DOI:** 10.1186/s13102-023-00723-2

**Published:** 2023-11-10

**Authors:** Juan Arnal-Burró, Carlos Vidal-Fernández, Cristina Igualada-Blazquez, Manuel Cuervas-Mons, Álvaro Martínez-Ayora, Alex Dos Santos-Vaquinhas

**Affiliations:** 1 Department of Orthopaedic Surgery, University Hospital Gregorio Marañón, C/ Doctor Esquerdo 46, Madrid, 28007 Spain; 2 Department of Orthopaedic Surgery, Torrejón University Hospital. C/ Mateo Inurria, Torrejón de Ardoz, Madrid, 28850 Spain

**Keywords:** Endurance sports, Runner’s knee, Iliotibial band syndrome, Arthroscopic knee surgery, Percutaneous lengthening of the iliotibial band

## Abstract

**Introduction:**

Description of a new surgical procedure (percutaneous lengthening and arthroscopic release, PLAR) that combines all the possible interventions on the iliotibial band (ITB), and evaluates its outcomes in a group of distance runners diagnosed with ITBS.

**Methods:**

A prospective observational study was made of distance runners diagnosed with ITBS and operated upon using the PLAR technique between 1 and 2018 and 31 June 2020. The surgical technique is described in detail, and the demographic data and functional outcomes measured by the sports performance scales Activity Rating Scale (ARS) and International Knee Documentation Committee (IKDC) are presented.

**Results:**

A total of 14 patients were included, with a mean follow-up of 16 months (range 12–42 months). All the patients resumed their previous sporting activity after an average of 4 (range 2.5-6) months, and no complications were recorded. In all cases, statistically significant improvement was evidenced by the ARS and IKDC scales following PLAR (p < 0.001), with excellent outcomes in 71% of the cases according to the ARS scale and in 86% according to the IKDC scale (mean difference between preoperative and final follow-up scores of 12.1/16 and 34.2/100 points, respectively).

**Conclusion:**

The PLAR technique is effective in allowing a return to previous sports performance levels in a short period of time among patients with ITBS refractory to conservative management, with a high satisfaction rate and the absence of complications.

## Introduction

Iliotibial band syndrome (ITBS) was first described by Renne in 1975 in recruits of the United States Marine Corps [1]. It is currently considered to be the second most common injury and the leading cause of lateral pain in the knee among runners (incidence 1.6–12%); it is thus also known as runner’s knee, though it is common in other sports such as cycling [2–4]. The classic presentation of ITBS is in the form of pain close to the lateral femoral condyle (LFC), manifesting after repetitive flexion-extension of the knee [3].

Anatomically, the iliotibial band (ITB) is a lateral thickening of the fascia lata of the thigh that inserts in Gerdy’s tubercle. In its distal portion, the ITB adheres to the intermuscular septum and is closely related to the LFC, displacing at 20–30º of flexion from anterior to a posterior location immediately above the condyle [5]. Although the origin of ITB syndrome is multifactorial, repetitive friction of the distal portion of the ITB against the LFC during 30° of flexion is the direct cause of inflammation of the lateral synovial recess, which when maintained over time gives rise to this syndrome [6].

Iliotibial band syndrome is initially treated on a conservative basis, with measures ranging from the modification of sports activity, to local corticosteroid (CS) injection, and local physiotherapy and muscle training. Firstly, these patients should include training modification, increasing the muscle training sessions to the detriment of continuous running, a common error in runners. The temporary clinical improvement afforded by corticosteroid and anaesthetic infiltration is quite constant in ITBS, for although the underlying aetiology is multifactorial, in most cases a local inflammatory process is found at LFC level [7]. Likewise, focal shock waves have been shown to be useful in chronic cases in which other physiotherapy techniques have failed [8].

Surgery would be reserved for high-performance athletes requiring rapid resolution of the symptoms and for those patients with persistent pain and limited activity for more than 6 months despite adequate adherence to well-designed conservative treatment [9]. Of the multiple surgical possibilities, the less invasive options seem to be gaining importance, as they allow to perform the same procedures as open surgery with the evident advantages afforded by minimally invasive surgery: less soft tissue aggression, less pain, less blood loss and shorter hospital stay. Furthermore, it offers possibility of performing a diagnostic examination allowing any concomitant lesions to be treated simultaneously [4, 6]. Open surgery include the resections initially described by Nobel [10], Martens et al. [11], Drogset et al. [12] and Holmes et al. [13], or more recent techniques such as Z-plasty lengthening [14] and bursectomy in cases of recalcitrant ITBS [15]. Less invasive options include percutaneous lengthening of the ITB, arthroscopic debridement of the lateral synovial recess [4, 16, 17], and a combination of both [18].

To our knowledge, no previous study has prospectively evaluated the results of the combined percutaneous and arthroscopic surgical management in a series of patients with ITBS. The purpose of the present study is to describe a novel surgical technique that combines percutaneous lengthening of the ITB and arthroscopic debridement of the lateral synovial recess (PLAR), and to evaluate its short term results in distance runners diagnosed with ITBS.

Our hypothesis is that PLAR could offer a high rate of return to the previous sports performance levels in cases of ITBS refractory to conservative treatment, with a lower rate of complications than open surgery.

## Materials and methods

### Study design

A prospective case series study was performed between 01/01/2018 and 31/06/2020. All patients gave informed consent to participation in the study, which was conducted in accordance with institutional standards.

### Patient population

The patients were enrolled consecutively. The inclusion criteria were all adult distance runners diagnosed with iliotibial band syndrome and with negative response to nonoperative treatment after six months. Distance runner was defined as professional or amateur patient running medium (1500 m) and long (marathon and ultra-trail runners) distances.

The exclusion criteria were: (i) incomplete clinical reports; (ii) non-distance runners; (iii) concomitant injuries interfering with running; (iv) bilateral involvement, (v) negative local anaesthetic infiltration test; and (vi) revision surgeries after previous ITB procedures.

Patient should meet all the inclusion criteria and none of the exclusion criteria. Before being included in the study, all patients performed a preoperative protocol, regardless of the complementary tests performed up to that time.

### Preoperative protocol

Complete medical history and physical examination were recorded in all patients. A local anaesthetic infiltration test was performed, which consisted in an ultrasound-guided sub-iliotibial bursa infiltration with 2ml of 2% mepivacaine, followed immediately by a 5 km race. If the patient´s symptoms were relieved temporarily during the race, the test was regarded positive.

**Fig. 1:**
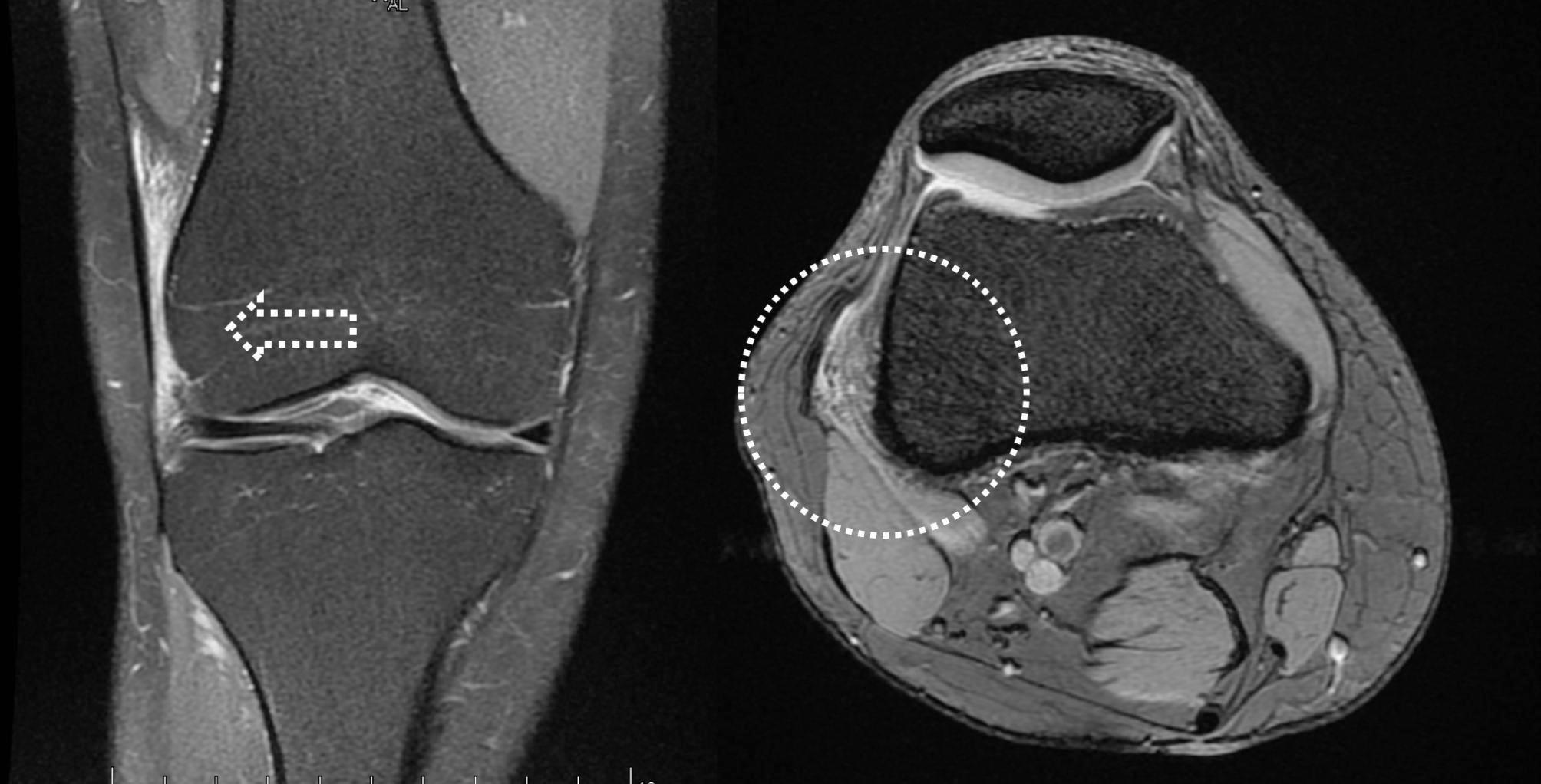
Preoperative MRI: coronal (right) and axial (left) views showing edema at the level of the ITB.

High-field MRI (≥ 1.5 T) was performed in all cases after sports had been performed by the patient in the 72 h before the scan, thereby increasing the sensitivity of the imaging technique when oedema appeared at the level of the LFC or ITB (Fig. 1).

Prior to the surgical indication, a specific rehabilitation program was conducted to optimize conservative management with those techniques that had not been yet applied in the patient, including stretches of the fascia lata, proximal eccentric muscle training, intratissue percutaneous electrolysis and at least three focal shockwave sessions.

### Independent and outcome variables

Demographic data (age, gender, and body mass index -BMI-), comorbidities, athletic discipline, time to surgery, and postoperative follow-up time were collected in all patients.

The intraoperative characteristics (time of ischemia, confirmation of ITBS, identification of concomitant lesions, and need for drainage) and intra- and post-operative complications were also recorded.

The main variables of the study were the rate and timing of return to previous sporting level, which were reported by patients at follow-up visits. Return to previous sport level was treated as a dichotomous outcome, and was defined as competing after undergoing the PLAR technique in at least one race of the same distance as pre-injury at or above the pre-injury level of competition. The return to sport rate was calculated from the number of athletes who returned to sport, out of the number of athletes who underwent the PLAR technique, and expressed as a percentage.

The secondary variables were the clinical evaluation of the patients based on the Activity Rating Scale (ARS), the International Knee Documentation Committee (IKDC) questionnaire, and the degree of satisfaction. The results of the ARS and IKDC scales were interpreted as follows: excellent = 95–100 for IKDC and 15–16 for ARS; good = 84–94 for IKDC and 13–14 for ARS; and fair = 65–83 for IKDC and 10–12 for ARS. The degree of satisfaction was evaluated in all patients with a poll based on the question: does the surgery meet your expectations?. The possible answers were: completely satisfied, mostly satisfied, somewhat satisfied, dissatisfied.

### Surgical procedure

**Fig. 2:**
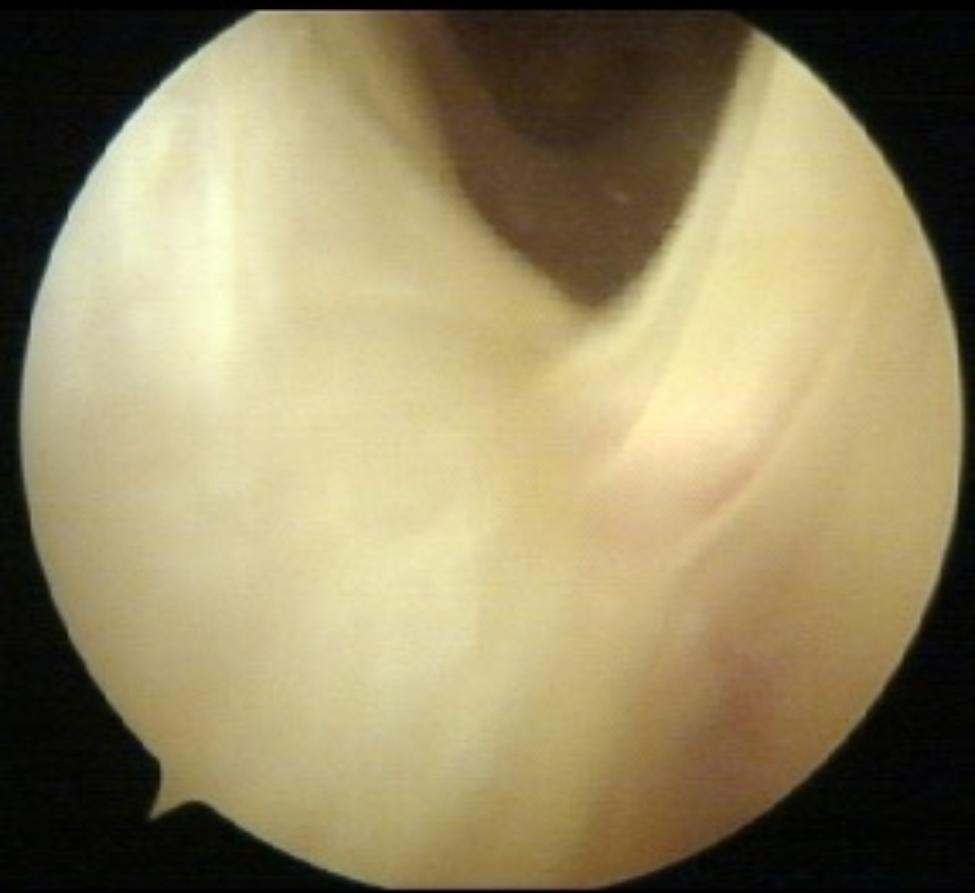
Intraoperative image. Fibrotic adhesions between the LFC and the ITB.

All procedures were performed by the same surgeon. The ITBS diagnosis was confirmed intraoperatively by observing a collapse of the space between the LFC and the ITB due to a mixture of bursitis and hard fibrotic adhesions preventing passage of the arthroscopy optics (Fig. 2).

The patients were placed supine on a conventional table with arthroscopy support, fitting an ischemia cuff to the thigh and performing standard aseptic preparation. The LFC, fibular head, Gerdy’s tubercle, and the anteromedial (AM) and anterolateral (AL) standards portals were identified and marked.

**Fig. 3:**
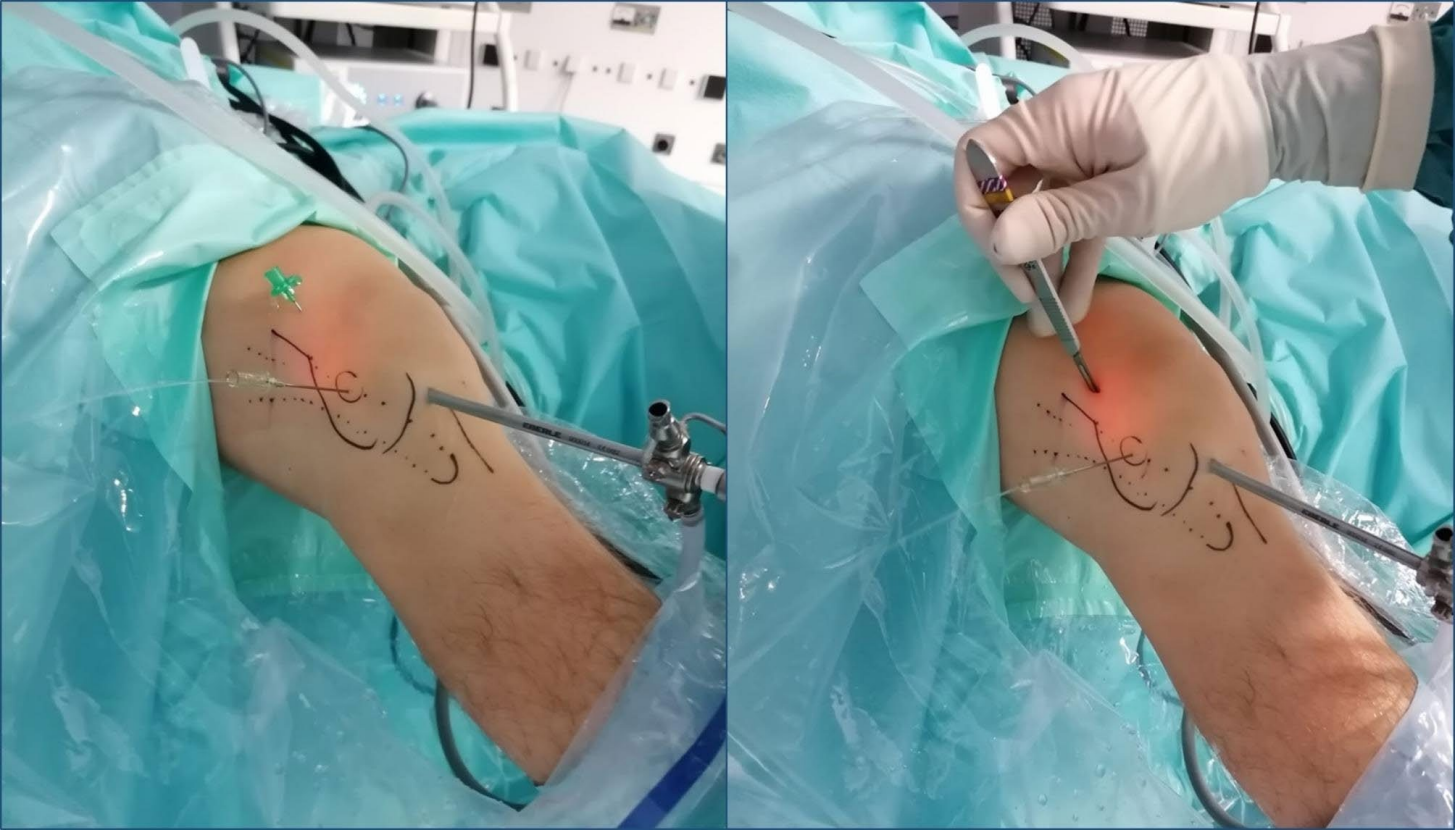
Intraoperative image. Superolateral portal (SLP) using a 16G Abbocath spinal needle as a guide

The procedure started with routine diagnostic arthroscopy through the AL portal. If there were doubts about concomitant lesions, an additional AM portal was used in order to be able to perform tactile examination of the knee structures. Under direct intraarticular vision, the superolateral (SL) portal was prepared using a 16G Abbocath spinal needle (Hospira, Lake Forest, IL, USA) as a guide, always through the tendinous portion of the vastus lateralis muscle or the capsule, making sure not to perforate the quadriceps muscle tissue (Fig. 3). All the portals were prepared with a No. 11 scalpel blade.

**Fig. 4:**
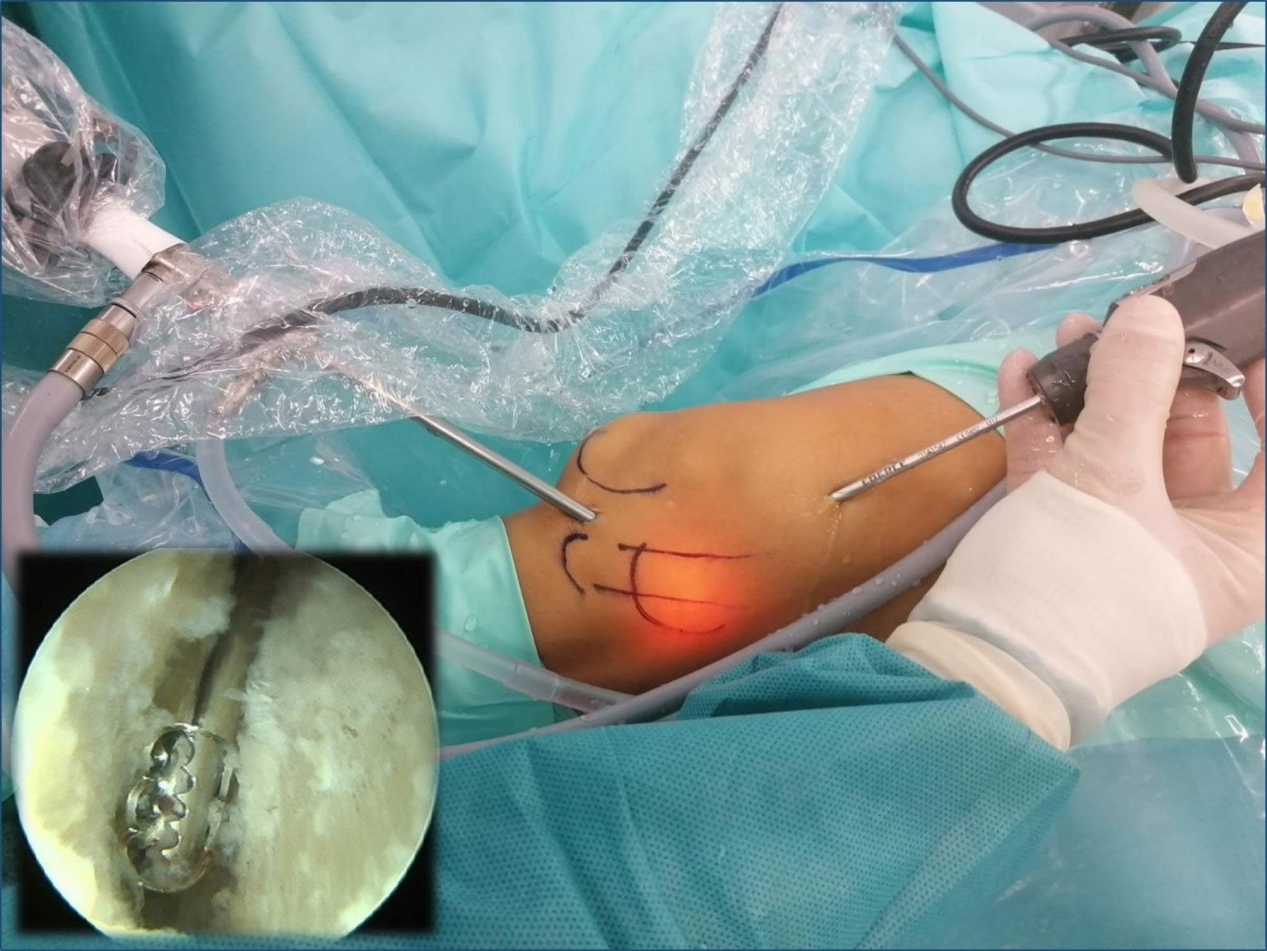
Intraoperative image. Release of the fibrous adhesions in the space between the LFC and ITB using a motorized shaver

**Fig. 5:**
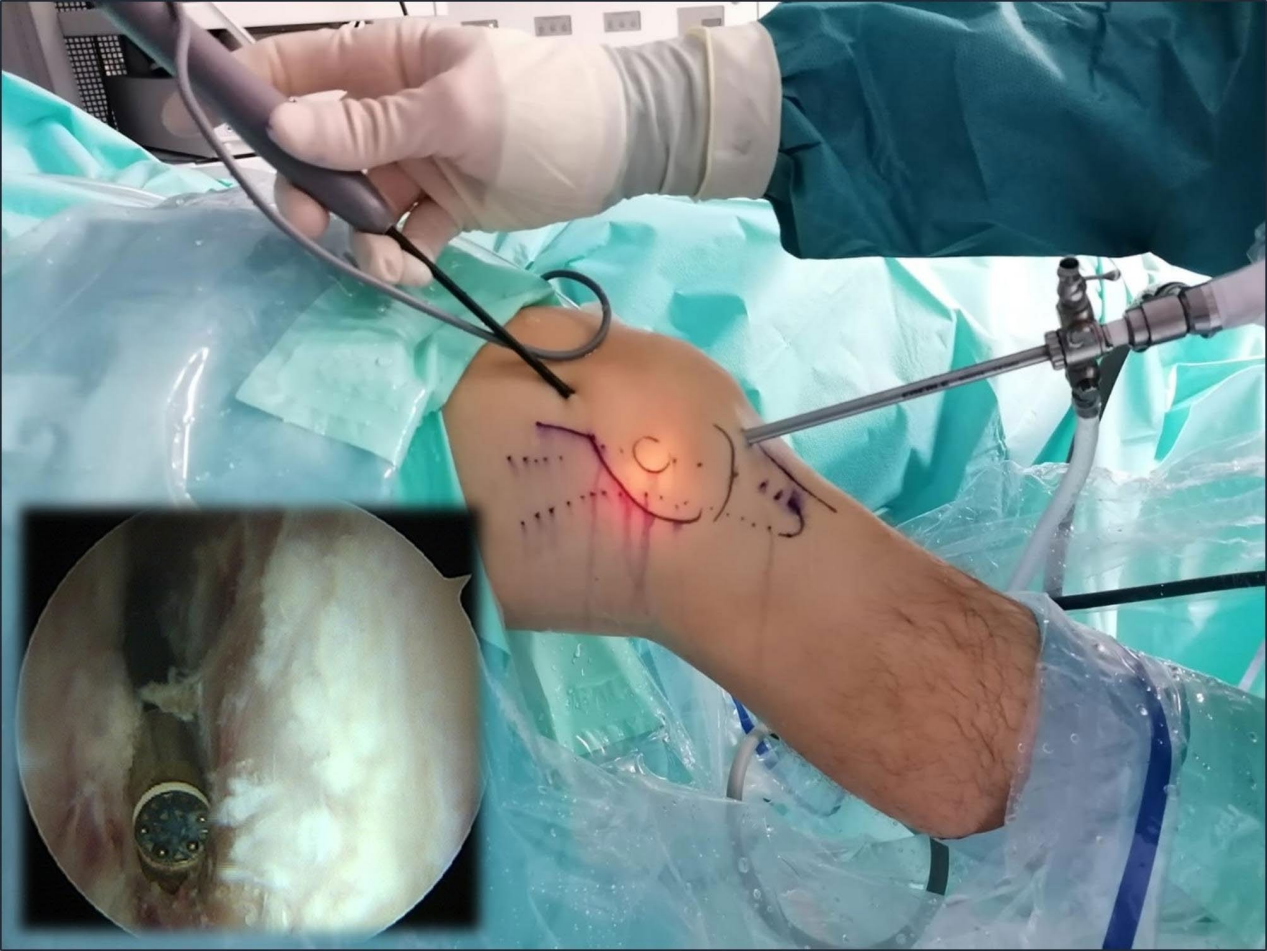
Intraoperative image. Release of the fibrous adhesions in the space between the LFC and ITB using a vaporizer

With the knee in 30º flexion we initially performed a debridement and resection of the lateral synovial recess, using a motorized shaver (Fig. 4) and a vaporizer (90-degree, model 405Q3, Bonss Medical Tech, Taizhou, Jiangsu, China) (Fig. 5). In patients with ITBS we can observe an abnormal anatomy with increased fibrosis in the lateral synovial recess, so we consider paramount to perform a wide resection in this area until we reach a complete view of the iliotibial band externally and the LFC medially, even seeing the external meniscal wall in its anterior half, and being able to advance the optics through from anterior to the popliteal tendon in the posterior zone, always preserving the meniscus-tibial and meniscus-femoral ligaments. This procedure was performed mainly from the SL portal under visual control from the AL portal, with inversion of the two portals to complete the release.

**Fig. 6:**
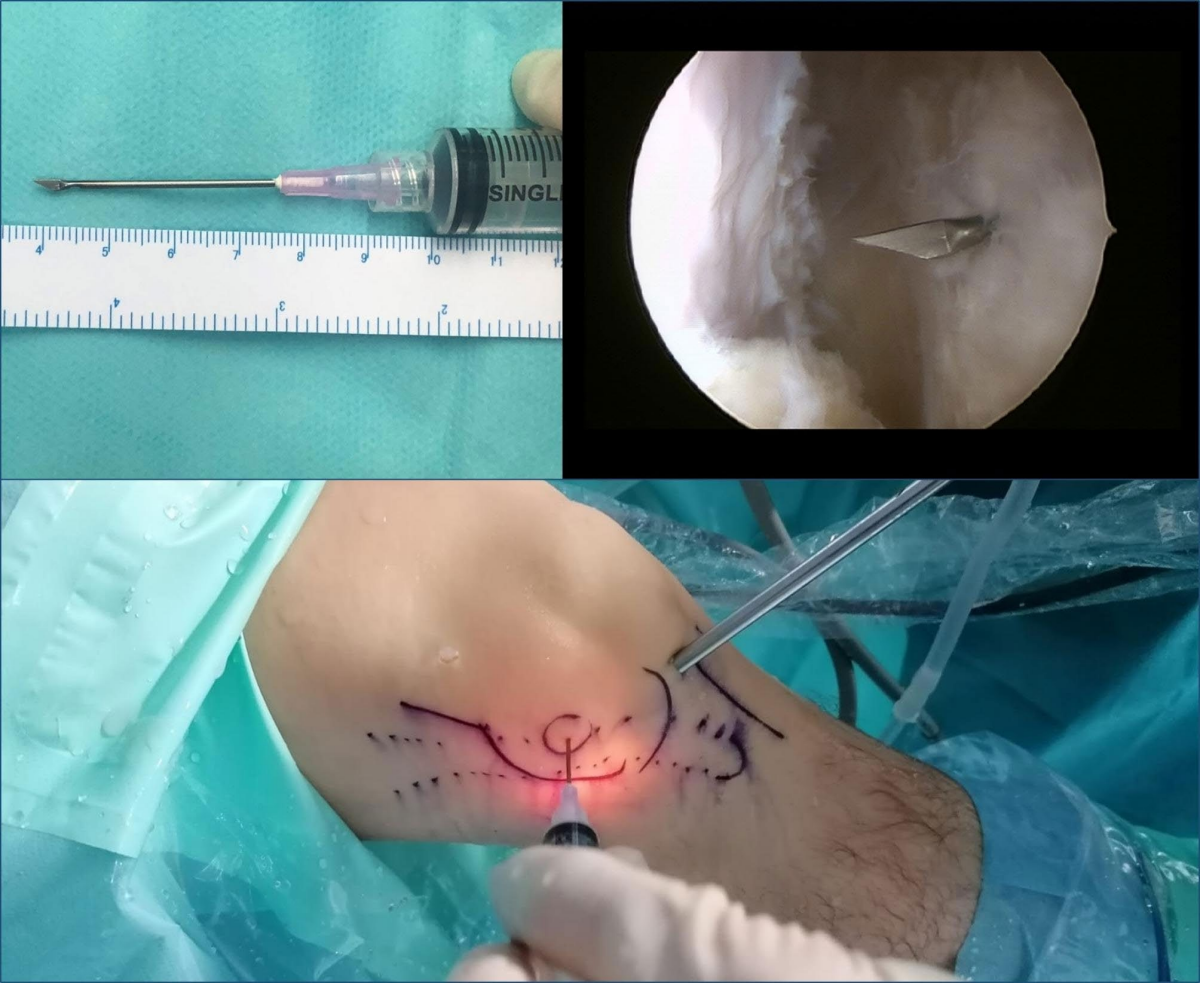
Intraoperative image. Micro-tenotomies on the ITB by an 18G 3-mm needle scalpel

The second part of the procedure involved percutaneous lengthening of the ITB under direct vision by arthroscopy. This was done with controlled knee varus at 30° of flexion, seeking a balance between lengthening and the preservation of muscle function. An 18G 3-mm needle scalpel (Nokor needle; Becton Dickinson and Co., Franklin Lakes, NJ, USA) was used to perform controlled micro-tenotomies as a micro-pie-crusting technique on the ITB. In all cases they were made longitudinal and parallel to the fibers, and in those cases with greater fibrosis of the ITB, the tenotomies were also made transversely in the posterior third (Fig. 6).

After completing the procedure, skin closure was performed with Prolene (Ethicon, Inc.) 2/0, and a compressive elastic bandage was placed, with a semirigid support in the external zone, where a bulge characteristically forms due to fluid extravasation through the micro-tenotomies. Redon drainage (Fresenius Kabi AG, Bad Homburg, Germany) was used for 12 h in patients with intraoperative identification of a sub-iliotibial bursa associated to significant vascular infiltration, and in all cases, we infiltrated a mixture of corticosteroids and local anesthetic (2 ml of Celestone Cronodose + 4 ml of 2% mepivacaine).

### Post-operative protocol

All patients were discharged with full weight-bearing assisted by two crutches according to tolerance.

Rehabilitation started from the first postoperative day. During the first two weeks, full joint range recovery exercises, isometric exercises and even post-supported squats were allowed in order to minimize muscle atrophy. Between weeks 2 and 4, eccentric muscle training (free, weight-bearing and single-foot squats, as well as frontal and lateral lunge exercises) combined with proprioception exercises using a BOSU (both-sides-up) ball or unstable platform were allowed. From weeks 4 to 8, plyometric exercises, elliptical tape, and static bicycle exercises were enhanced, and gentle skipping exercises were allowed, according to tolerance. From the 8th week, and depending on the muscle and proprioceptive condition of the patient, we allowed running a distance of 1 km every other day, combining walking and running stretches, and added distance or speed increments of 10% every two days if tolerance proved good. From the 12th week after surgery, recovery was authorized to continue at the athletics club under the control of the coach or physiotherapist.

### Follow-up protocol

A minimum follow-up of 12 months was performed. Postoperative data were collected in all patients at 15 days, 1, 3, 6 and 12 months, and at the end of follow-up (medical discharge). Complications and clinical course were assessed at all visits, while the sporting performance and the ARS and IKDC questionnaires were assessed at 3, 6 and 12 months, without access to a copy of the scales during the interim period, in order to prevent patient self-monitoring of recovery and influencing the final outcome. The degree of satisfaction was recorded at the last follow-up visit.

### Statistical analysis

The statistical analysis was performed using SPSS® version 22.0 package for Mac (IBM, NY, USA). Statistical significance was considered for p ≤ 0.05 and a statistical power of 90%.

Standard descriptive statistics including measures of central tendency (mean/median) and variance (standard deviation [SD]/interquartile range [IQR]) were calculated, as well as frequencies and proportions.

The preoperative and final follow-up functional scores were compared using the Wilcoxon signed-rank test.

A multiple non-parametric analysis comparing the IKDCS and ACS scales preoperatively and at 6 and 12 months was performed using the Friedman’s statistical test.

## Results

An initial cohort of 26 patients with a diagnosis of chronic ITBS were referred during the study period. A total of 12 patients were excluded, 5 due to a negative local anaesthetic infiltration test, 6 for improving after a modification of the rehabilitation program previously performed in less than 6 months, and 1 due to previous surgery, resulting in a final sample of 14 patients.

### Demographic data

The mean age of the patients was 27 (range 17–38) years, 12 (86%) men, and 2 (14%) women, with a mean BMI of 21.2 (± 0.9) kg/m^2^. No patient had medical comorbidities. The practiced athletics discipline were: 1500 m 3 patients (21%), marathon 8 patients (57%), and ultra-trail runners 3 patients (21%). A statistical description of the results is depicted in Table 1.

**Table 1:** Demographic, diagnostic and intraoperative data

Age	27 (r 17–38) years
Sex	
Male Female	12 (86) 2 (14)
BMI	21.2 (SD ± 0.9) kg/m^2^
Athletic discipline	
Ultra-trail Marathon 1500 m	3 (21) 8 (57) 3 (21)
Time to surgery Follow-up time	16 (r 7–41) months 16 (r 12–33) months
Ischemia time	48 (r 37–63) minutes
Concomitant lesions	
Type I/II PF chondropathy Drainage	5 (36) 5 (36)

Values are n (%), mean ± SD or r unless otherwise noted

*Abbreviations: r* range, *SD* standad deviation, US ultrasound, *PF patellofemoral*

The mean time to surgery since the first visit was 16 (range 7–41) months, and the mean postoperative follow-up period was 16 (range 12–33) months.

### Intraoperative data

The mean ischemia time was 48 (range 37–63) minutes. In all patients, involvement of the ITB was confirmed intraoperatively, and concomitant lesions were ruled out, except for grade I/II patellofemoral chondral lesions in 5 (36%) patients. Five (36%) patients required postoperative Redon drainage (Table 1).

### Clinical outcomes

#### Main variables

All the patients resumed their previous sporting activity after an average of 4 (range 2.5-6) months (Table 2).

**Table 2:** Clinical outcomes and complications

Return to previous sport level	
Rate Time	14 (100) 4 (r 2.5-6) months
ARS	
Preoperative 3 months 6 months 12 months FFU	2.9 (r 1–5) 3 (r 1–5) 14.8 (r 12–16) 15 (r 12–16) 15 (r 12–16)
Excellent Good	11 (79) 3 (21)
FFU - preoperative 6 months - preoperative 12 months − 6months	12.1 (p 0.000^*^) 11.9 (p 0.01^*^) 0.2 (p > 0.05)
IKDC	
Preoperative 3 months 6 months 12 months FFU	63.1 (r 52–75) 62.4 (r 55–70) 96.9 (r 92–100) 97.1 (r 92–100) 97.3 (r 92–100)
Excellent Good	13 (93) 1 (7)
FFU - preoperative 6 months - preoperative 12 months − 6months	34 (p 0.000^*^) 33.8 (p 0.01^*^) 0.2 (p > 0. 05)
Degree of satisfaction	
Completely satisfied Mostly satisfied Somewhat satisfied Dissatisfied	12 (86) 2 (14) 0 0
Complications	0

Values are n (%), mean ± SD or r unless otherwise noted

*Abbreviations: r* range, *FFU* final follow-up

* p < 0.05

#### Secondary variables

The mean improvement in the ARS scale was 12.1 points (p < 0,001), from 2.9/16 (range 1–5) points preoperatively to 15/16 (range 12–16) points at final follow-up, with 11 (79%) patients achieving an excellent outcome, and 3 (21%) a good outcome. The mean improvement in the IKDC scale was 34 points (p < 0,001), from 63.1/100 (range 52–75) points preoperatively to 97.1/100 (range 92–100) points at final follow-up, with 13 (93%) patients achieving an excellent outcome, and 1 (7%) a good outcome.

Multiple non-parametric analysis showed a statistically significant improvement in the ARS and IKDC scales at month 6 compared to preoperatively (11.9/16 and 33.8/100 points respectively; p = 0.01), and a non-statistically significant improvement at month 12 compared to month 6 (0.2/16 and 0.2/100 points respectively; p > 0.05).

At the end of follow-up, 12 (86%) patients claimed to be completely satisfied with the outcome of surgery, and two (14%) were mostly satisfied.

No systemic or local complications were recorded in any patient.

## Discussion

Iliotibial band syndrome is a common cause of lateral knee pain in athletes. The diagnosis is eminently clinical, based on a detailed history and physical examination. Ultrasound and MRI may be useful tools for differential diagnosis and postsurgical monitoring [5, 9, 19, 20]. However, the use of MRI to confirm the diagnosis is more controversial, and in our study we detected two problems with this test. Firstly, according to our data, MRI yielded a false negative rate of 29%, despite the fact that its sensitivity was tried to be increased by performing physical exercise in the 72 h prior to the scan. Secondly, we observed differential misclassification bias with the use of MRI, since the radiologist tended to report the test as suggestive of ITBS despite the mere presence of nonspecific changes such as an increased thickness of the ITB or increased fluid in the external recess, when a clinical suspicion of ITBS was stated in the request for the MRI study. In this regard, we consider ultrasound-guided local anaesthetic infiltration before a running test to be particularly important in the diagnostic algorithm.

ITBS is initially treated on a conservative basis. However, this management has been shown to effectively reduce the symptoms during a maximum follow-up time of 6 months [6]. In patients refractory to such treatment, orthopaedic surgeons should be able to offer an alternative, particularly for those individuals whose professional activity depends directly on their running capacity. This is the case of the patients reported in our study, which included individuals pending a university scholarship in relation to their athletic performance, and others preparing physical tests for access to different national security forces.

There are multiple surgical options, with a return to sports rate of 81–100% [6]. Despite the good results reported with open surgery [10, 11, 13–15], we consider it essential to be able to offer minimally invasive surgery to our patients, due to the evident advantages of this procedures. According to the results of our study, PLAR appears to achieve this objective, with a rate of return to previous sports activity of 100%, and no local or systemic complications recorded. Moreover, our study showed statistically significant improvement in the ARS and IKDC functional scores, with a mean difference between the final follow-up and the preoperative visits of 12.1/16 and 34/100 points, respectively. The main improvement was seen in the first 6 months after surgery, with a statistically significant difference in both scales compared to the preoperative score (11.9/16 and 33.8/100 points, respectively). This improvement was maintained at 12 months and even increased (0.2/16 and 0.2/100 points, respectively), this time non-significantly. Excellent outcomes were also obtained in 79% of the cases with the ARS scale, and in 93% with the IKDC scale, having all other patients good outcomes. In addition, 86% of the patients claimed to be completely satisfied after surgery, and 14% were mostly satisfied.

Results consistent with those of our own study have been previously reported in the literature. Michels et al. [16] presented the results of a series of 33 patients subjected to debridement of the lateral synovial recess using a totally intraarticular arthroscopic technique. All patients were able to perform slow running three months after the operation, 80% reported excellent outcomes, and 17% good outcomes, based on the functional scale of Drogset et al. [12] Cowden and Barber [4] described a similar procedure in a single 41-year-old male who ran marathons and was able to return to athletic activity without discomfort after the pain disappeared four weeks following surgery. However, none of the previous studies used a combined arthroscopic and percutaneous technique for sub-iliotibial release and ITB elongation, representing global management of ITBS. Pierce et al. [18] described a combined technique adding arthroscopic lengthening of the ITB through incisions with Metzenbaum scissors at proximal, distal, anterior and posterior level of the ITB, from its point of insertion in Gerdy’s tubercle. However, to our knowledge, no previous studies have evaluated the results of arthroscopic management combining both techniques in a prospective series of patients.

Regarding the surgical procedure, in the first cases we used intraoperative ultrasound support to check the position of the needle scalpel and confirm total release of the ITB. However, this measure was subsequently removed from our protocol, as it added surgery time and offered no technical advantage over direct intraarticular visualization, which allows us to check the mechanical properties of the ITB through direct palpation, and confirm its increased elasticity after the micro-tenotomy-induced release.

## Limitations

This study has several limitations, including its small sample size. This, and the fact that all patients were distance runners, could limit extrapolation of the results to the general population. However, ITBS is practically exclusive to athletes. We therefore considered that it would be more significant to conduct a specific analysis of the results of our technique in this population subgroup. On the other hand, the minimum follow-up time of 12 months could also be considered a limitation. Nevertheless, the choice of this time period was based on sports performance recovery criteria after consulting coaches with experience in rehabilitation. The mentioned follow-up time was thus considered suitable for assessing recovery of the previous sports level in the absence of complications. Lastly, it could be considered that there are more complete knee functional assessment scales than those used in our study. However, we decided to use the ARS and IKDC scales because there was little clinical impact upon the daily activities of the patients included in the study. We thus considered that we needed more specific scales to adequately assess the sports performance impact of ITBS and improvement after the operation.

## Conclusion

Based on the results obtained, it can be concluded that the PLAR technique is effective in allowing a return to previous sports performance levels in a short period of time among patients with ITBS refractory to conservative management, with a high satisfaction rate and the absence of complications.

## Data Availability

The datasets generated and/or analysed during the current study are not publicly available due to confidentiality reasons, but are available from the corresponding author on reasonable request.
